# Predicting Attrition in a Text-Based Nutrition Education Program: Survival Analysis of Text2BHealthy

**DOI:** 10.2196/mhealth.9967

**Published:** 2019-01-21

**Authors:** Stephanie K Grutzmacher, Ashley L Munger, Katherine E Speirs, Yassaman Vafai, Evan Hilberg, Erin Braunscheidel Duru, Laryessa Worthington, Lisa Lachenmayr

**Affiliations:** 1 School of Biological and Population Health Sciences College of Public Health and Human Sciences Oregon State University Corvallis, OR United States; 2 Department of Child and Family Studies California State University, Los Angeles Los Angeles, CA United States; 3 Department of Family Studies and Human Development Norton School of Family and Consumer Sciences University of Arizona Tucson, AZ United States; 4 Department of Family Science School of Public Health University of Maryland College Park, MD United States; 5 Maryland Food Supplement Nutrition Education University of Maryland, College Park Columbia, MD United States

**Keywords:** text messaging, retention, diet, food, and nutrition, food assistance, parents, survival analysis

## Abstract

**Background:**

Text-based programs have been shown to effectively address a wide variety of health issues. Although little research examines short message service (SMS) text messaging program characteristics that predict participant retention and attrition, features of SMS text message programs, such as program duration and intensity, message content, and the participants’ context, may have an impact. The impact of stop messages—messages with instructions for how to drop out of an SMS text message program—may be particularly important to investigate.

**Objective:**

The aim of this study was to describe attrition from Text2BHealthy, a text-based nutrition and physical activity promotion program for parents of low-income elementary school children, and to determine the impact of message content and number of stop messages received on attrition.

**Methods:**

Using data from 972 parents enrolled in Text2BHealthy, we created Kaplan-Meier curves to estimate differences in program duration for different SMS text message types, including nutrition, physical activity, stop, and other messages. Covariates, including rurality and number of stop messages received, were included.

**Results:**

Retention rates by school ranged from 74% (60/81) to 95.0% (132/139), with an average retention rate of 85.7% (833/972) across all schools. Program duration ranged from 7 to 282 days, with a median program duration of 233 days and an average program duration of 211.7 days. Among those who dropped out, program duration ranged from 7 to 247 days, with a median program duration of 102.5 days. Receiving a stop message increased the probability of attrition compared with receiving messages about nutrition, physical activity, or other topics (hazard ratio=51.5, 95% CI 32.46-81.7; *P*<.001). Furthermore, each additional stop message received increased the probability of attrition (hazard ratio=10.36, 95% CI 6.14-17.46; *P*<.001). The degree of rurality also had a significant effect on the probability of attrition, with metropolitan county participants more likely to drop out of the program than rural county participants. The interaction between SMS text message type and total number of stop messages received had a significant effect on attrition, with the effect of the number of stop messages received dependent on the SMS text message type.

**Conclusions:**

This study demonstrates the potential of SMS text message programs to retain participants over time. Furthermore, this study suggests that the probability of attrition increases substantially when participants receive messages with instructions for dropping out of the program. Program planners should carefully consider the impact of stop messages and other program content and characteristics on program retention. Additional research is needed to identify participant, programmatic, and contextual predictors of program duration and to explicate the relationship between program duration and program efficacy.

## Introduction

### Background

Using text messages, also known as short message service (SMS), to deliver or supplement health interventions has increased in popularity in recent years. SMS text messages are an appealing mode of program delivery largely because they can be easily and inexpensively used to reach a broad audience [1]. In the United States, approximately 95% of adults own a cell phone [2] and 81% of US adults with cell phones use SMS text messages [3]. SMS text messages may also be a useful tool for accessing underserved populations and addressing health disparities. Low-income and minority populations use cell phones at rates that equal or exceed those of their higher-income and white counterparts [2].

Text-based programs have been successfully used to address a wide variety of health issues such as weight loss [4], smoking cessation [5], diabetes management [6], and sexual health [7]. Exposure to health-related SMS text messages has also been effective in promoting participants’ adherence to a program and maintaining healthy behavior changes [8]. A meta-analysis investigating the efficacy of SMS text message–based health promotion interventions found that the overall weighted mean effect size on health outcomes among the 19 randomized controlled trials included in analysis was *d*=0.329 (95% CI 0.274-0.385; *P*<.001), indicating a small effect across studies [9].

Although many studies have shown that retention is generally high in SMS text message programs [8,10-12], retention rates also vary quite widely. The aforementioned meta-analysis found that mean retention at follow-up among the included studies was 86% [9]. In another meta-analysis, which investigated the efficacy of weight management programs that incorporated SMS text messages, the retention rate at the postintervention stage among the 14 included studies ranged from 46% to 96% [13].

Understanding when and why participants tend to drop out of SMS text message programs is imperative for effective program planning. There is some evidence that when participants drop out, they do so early in the program; Coa and Patrick found that among those who dropped out of a diet and physical activity SMS text message program, 65% (54/83; 28% of all users) did so within the first 2 weeks [14]. As SMS text message programs vary widely in content, frequency, relevance, tone, theoretical underpinnings, and other characteristics, we do not know whether timing of dropout is related to these characteristics or whether participants are simply tired of SMS text message programming over time. Knowing typical timing of dropout can help researchers prioritize content delivery and develop evaluation plans that maximize time with the majority of participants. Though there are several factors that could potentially impact attrition, such as message content and frequency, these factors remain unexamined, making it difficult to provide guidance concerning program development and features that hinder retention.

### Objectives

The purpose of this study was to describe attrition from Text2BHealthy, an SMS text message-based healthy eating and physical activity promotion program. Due to the lack of existing research about attrition from SMS text message programs, certain features of Text2BHealthy thought to be most likely to influence attrition were selected for examination. For example, in the program, several types of message content were delivered, including messages about nutrition, physical activity, and *stop messages*. Stop messages provide participants with instructions for withdrawing from the program. Due to a concern of the program funding agency about participants without SMS text messaging in their data plans incurring costs to receive program messages, stop messages were a required feature of the program. It is unknown whether and how much such messages increase attrition. In addition, the Text2BHealthy program was tailored to particular elementary schools located throughout the state of Maryland. These schools varied widely in terms of the degree of rurality of the area. Participants in rural areas might be less likely to drop out of a text-based health promotion program as rural areas tend to have fewer health services and programs available [15].

In this study, we sought to discern (1) how long participants remained in the program before dropping out; (2) whether particular types of messages, particularly stop messages, increased the likelihood that participants will drop out of the program; (3) whether the number of stop messages received increased the likelihood that participants will drop out of the program; (4) whether school rurality was associated with attrition; and (5) whether the impact of the number of stop messages received differs by message type.

## Methods

### Text2BHealthy Program

Text2BHealthy is a Maryland Supplemental Nutrition Assistance Program-Education (SNAP-Ed) nutrition and physical activity promotion program delivered by SMS text messages to parents of elementary school children [16]. Parents received message content tailored to their children’s school and local community. Program participants received 2 to 3 messages per week. Messages provided information and actionable nudges about nutrition and physical activity as well as a variety of other related content. Although Text2BHealthy did not typically solicit responses to most messages, participants were occasionally asked to respond to simple evaluation questions or set goals via text response. Participants were able to remove themselves from the program at any time by texting *stop* to the program short code or responding to any message with a message that included *stop*. As required by the program funding agency, SMS text messages informing participants about how to leave the program were sent approximately every 6 weeks.

### Participants and Recruitment

Participants included in this study were 972 parents of children attending a selection of low-income elementary schools with youth SNAP-Ed programs in Maryland and participating in the Text2BHealthy program. Parents were recruited through school events and program promotional items sent home at the beginning of the school year. They were enrolled by either providing their phone number to program staff or enrolling themselves through a keyword texted to a short code. Data for this study came from the September 2012 to June 2013 program year, which includes enrolled parents from 10 elementary schools in 5 Maryland counties and Baltimore City. Parents could enroll in and drop out of Text2BHealthy at any point during the program year.

### Variables and Measures

The outcome of interest for this study was attrition from the Text2BHealthy program (ie, survival time in days). Attrition data were recorded by the Web platform that was used to send messages to participants. When participants sent an SMS text message to the program phone number indicating that they wanted to be removed from the program, the Web platform would automatically and instantly remove them from the list of participants to receive SMS text messages and record the date and time that they left the program. Program enrollment date and dropout date were used to calculate program duration (ie, survival time) in days.

Survival analyses were conducted to examine differences in participant attrition from the program. The primary predictor for survival time, in days, was message type. Message type was created by coding the last SMS text message received (either the last message before dropout or the last message sent during the program year) into 5 categories: nutrition; physical activity; stop messages describing how to drop out of the program; stop messages combined with content about nutrition or physical activity; and a variety of other content that included evaluation questions, goal-setting, and healthy event notifications and other messages not explicitly addressing nutrition and physical activity actions (see Table 1).

Several covariates were included in the models, including rurality and the number of stop messages received during the program year. Rurality was determined for each school using the United States Department of Agriculture’s 2013 Rural-Urban Continuum Codes (RUCC; the year the data were collected) for the county where the school was located. These codes range from 1 to 9, with 1 to 3 indicating metropolitan areas and 4 to 9 indicating nonmetropolitan areas [17].

### Statistical Analysis

Frequencies were used to determine retention rates. Mean and median program durations were calculated to determine how long participants remained in the program before dropping out. Kaplan-Meier curves were created to estimate differences in participant attrition for different message types. Additional models that included total number of stop messages received, RUCC, and interactions between message type and total number of stop messages received were run. Using the survival package, Cox proportional hazards models were fit to estimate hazard ratios. A graphical inspection of the residuals was done to test the proportional hazards assumption. Pairwise comparisons were conducted to determine the effect of the number of stop messages by message type. All analyses were run in R Statistical Computing Package (v 3.2.1, R Foundation for Statistical Computing).

**Table 1:** Message types, frequency, and examples of the last message received.

Message content type	Messages sent, n	Message example
Nutrition	587	Some students made mango salsa in nutrition class last week. All children have a copy of the recipe in their backpack today. Give it a try!
Physical activity	170	It’s going to be almost 50 degrees this afternoon! Enjoy some time outside with the kids after school. Take a walk with your family or play a game of catch!
Stop	55	Text2BHealthy is a free program from the University of Maryland. If this program does not fit into your text plan, or you no longer want messages reply STOP.
Nutrition with stop^a^	14	Broccoli is in season and $1.79 per pound at [local grocery store]. Steam or eat raw with low fat dip. Kids love broccoli! Msg & Data Rates May Apply. Reply STOP to quit.
Physical activity with stop^a^	16	It’s December & it’s warm outside! Children love the extra time to play outside before dinner. Msg & Data Rates May Apply. To quit receiving messages, reply STOP.
Other^b^	130	Take the family downtown this Saturday & Sunday for the Book Festival. Read, dance & hear from chefs & food experts.

^a^Nutrition with stop and physical activity with stop were combined because of the small number of messages in each category.

^b^Other message types represent a broad variety of content that could not be classified into 1 of the 5 predominant message content types. Other messages include evaluation questions and survey reminders, goal setting, and general community health event notifications.

## Results

### Descriptive Statistics

There were 972 participants during the 2012 to 2013 program year. Among the 10 participating schools, the average free and reduced meals (FARM) rate, indicating the percentage of students in the school receiving free or reduced price meals, was 80.9%. According to the 2013 RUCC [17], 7 out of 10 (70%) schools were located in metro counties, with populations of 1 million or more, whereas 1 school was located in a metro county with a population of fewer than 250,000, and 2 schools were located in a nonmetro county, with urban populations between 2500 and 19,999. Retention rates by school ranged between 74% (60/81) and 95.0% (132/139), with an average retention rate of 85.7% (833/972) across all schools. Program duration ranged from 7 to 282 days, with a median program duration of 233 days and average program duration of 211.7 days (see Table 2). In total, 14.3% (139/972) of participants dropped out of the program. Among those who dropped out, program duration ranged from 7 to 247 days, with a median program duration of 102.5 days.

### Survival Analysis

**Figure 1:**
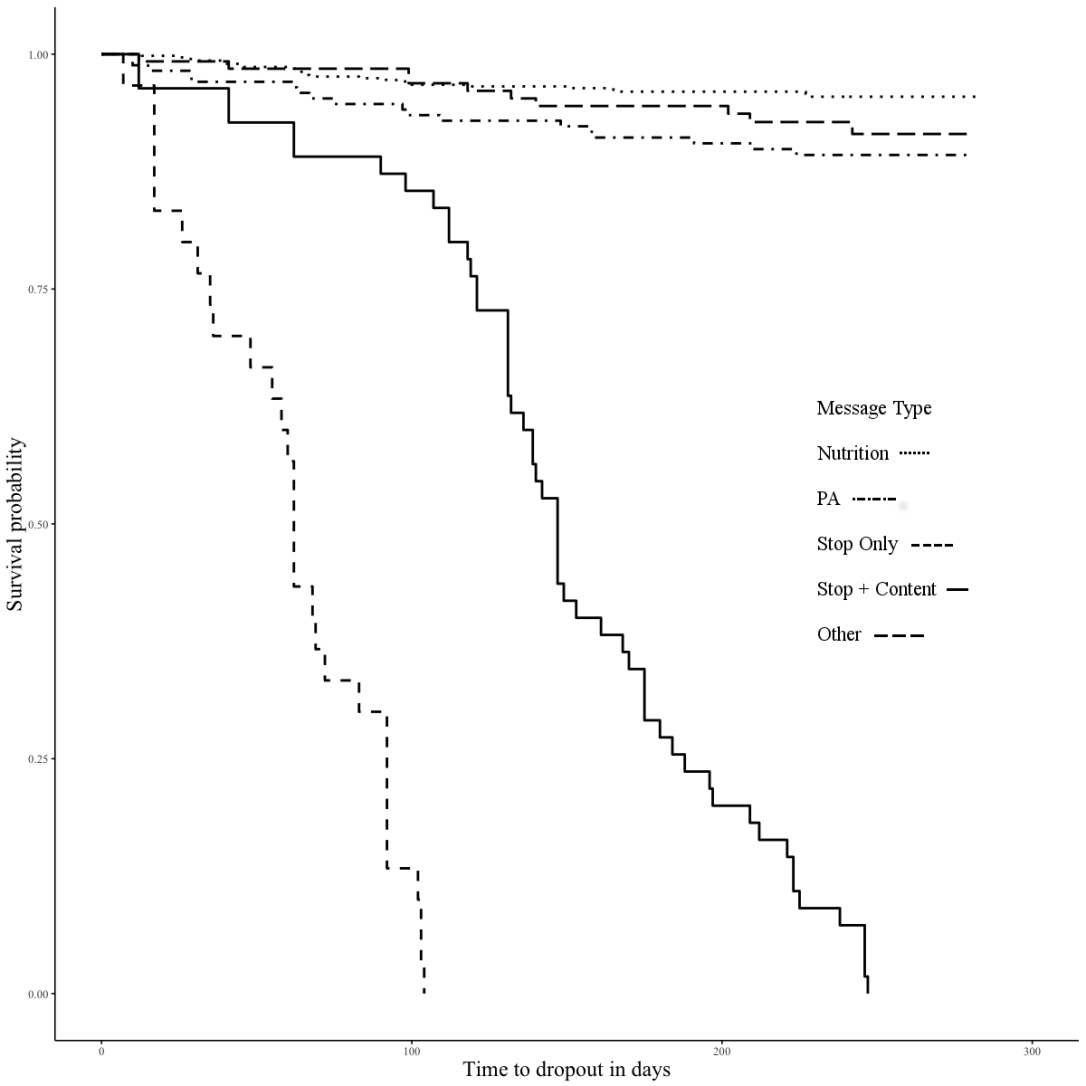
Kaplan-Meier survival curves by message type.

Kaplan-Meier survival curves indicate differences in program attrition by SMS text message type (see Figure 1). A log rank test was used to examine differences in attrition among SMS text message types (χ^2^_3_=916.6; *P*<.001). Among those who did drop out of the program, participants were more likely to drop out after having received a stop message, followed by a stop message paired with nutrition or physical activity content and then by physical activity content alone. Participants were least likely to drop out after receiving a nutrition message. On the basis of these findings, Cox proportional hazards models were fit to estimate the effect of several covariates on program attrition.

Model 1 examines the predictive effect of SMS text message type on program attrition (see Table 3). As seen in Figure 1, stop messages are associated with a high probability of program attrition (hazard ratio=51.5, 95% CI 32.46-81.7; *P*<.001). The addition of covariates to the model attenuates this relationship; however, the magnitude of the effect of stop messages remains substantial in comparison with nutrition messages. In model 2, which examines SMS text message type and the total number of stop messages received, the hazard ratio decreases substantially to 10.36 (95% CI 6.14-17.46; *P*<.001). In addition, model 2 shows that the more stop messages a participant receives, the greater the probability of attrition. In model 3, rurality of the program site impacts attrition, as the addition of the RUCC increases the hazard ratio to 11.60 (95% CI 6.78-19.84; *P*<.001).

**Table 2:** School characteristics.

School (n=participants)	FARM^a^ rate (%)	RUCC^b^	Retention rate (%)	Duration (days), mean (SD)	Duration (days), median
1 (participants, n=65)	97	1	86	202.31 (71.6)	234
2 (n=46)	63	6	85	223.93 (52.2)	241
3S^c^ (n=99)	77	1	83	198.42 (64.2)	219
3E^d^ (n=55)	77	1	80	198.91 (69.1)	219
4 (n=119)	91.2	1	84.9	215.45 (47.7)	233
5 (n=139)	77.7	3	86.3	213.12 (60.2)	243
6 (n=95)	99	1	97	229.38 (34.5)	240
7 (n=68)	75	1	85	183.25 (71.6)	226
8 (n=139)	92.4	1	95.0	230.14 (33.3)	240
9 (n=66)	73	1	76	195.03 (75.5)	221
10 (n=81)	63	6	74	213.89 (61.4)	227
Total (N=972)	80.9	—^e^	85.7	211.66 (58.8)	233

^a^FARM: free and reduced meals. Students are eligible for free school meals if household annual income falls below 130% of the federal poverty guidelines. Students are eligible for reduced price meals if household annual income falls between 130% and 185% of the federal poverty guidelines. FARM rates are school-level data that represent the entire population of each school, not the Text2BHealthy participant sample.

^b^RUCC: Rural-Urban Continuum Code. RUCC 1: fringe counties of metro areas of 1 million population or more (metro county); RUCC 3: counties in metro areas of fewer than 250,000 population (metro county); RUCC 6: urban population of 2500 to 19,999, adjacent to a metro area (nonmetro county).

^c^3S: Spanish language messages sent to participants at school 3. These 2 groups were kept separate because 3 participants from school 3 elected to receive both English and Spanish messages. These 2 groups were kept separate because 3 participants from school 3 elected to receive both English and Spanish messages.

^d^3E: English language messages sent to participants at school 3. These 2 groups were kept separate because 3 participants from school 3 elected to receive both English and Spanish messages.

^e^Not applicable.

Living in a metro county with populations under 250,000 (RUCC=3) is associated with higher probability of dropping out of the program (hazard ratio=4.27, CI 2.33-7.83; *P*<.001), whereas living in a nonmetro county with a population between 2500 and 19,999 people (RUCC=6) is associated with lower, but statistically insignificant probability of dropping out of the program (hazard ratio=0.63, CI 0.39-1.02; *P*>.05), respectively, compared with living in a metro county with populations greater than 1 million (RUCC=1).

In model 4, the final model in this analysis, interaction terms between the total number of stop messages and SMS text message types are included. The effect of the interaction term was explored through pairwise comparisons by message type (see Pairwise Comparisons by Message Type section). The addition of all the covariates and the interaction terms to the model leads to a 99% decrease in the effect of stop message only from model 1, and the association is no longer statistically significant (*P*>.05). The interaction between stop messages and the total number of stop messages received, however, has the largest effect on likelihood of dropping out of the program (hazard ratio=3.31, 95% CI 2.35-4.64; *P*<.001), suggesting a possible moderating effect of the total number of stop messages on the association between receiving stop messages and attrition rate in the program.

**Table 3:** Predictors of attrition by message type.

Variable^a^	Model 1^b^ (95% CI)	*P* value	Model 2^c^ (95% CI)	*P* value	Model 3^d^ (95% CI)	*P* value	Model 4^e^ (95% CI)	*P* value
Physical activity message	2.50 (1.36-4.58)	.003	0.76 (0.39-1.47)	.41	0.88 (0.45-1.74)	.71	0.17 (0.06-0.49)	.001
Stop message	51.5 (32.46-81.7)	<.001	10.36 (6.14-17.46)	<.001	11.60 (6.78-19.84)	<.001	0.43 (0.16-1.16)	.10
Other message	1.82 (0.87-3.80)	.11	1.00 (0.46-2.22)	.98	0.45 (0.19-1.08)	.07	0.21 (0.04-1.08)	.06
Total stop messages received	—^f^	—	0.04 (0.02-0.05)	<.001	0.03 (0.02-0.04)	<.001	0.02 (0.01-0.03)	<.001
County RUCC^g^ of 3	—	—	—	—	4.27 (2.33-7.83)	<.001	3.05 (1.65-5.64)	<.001
County RUCC of 6	—	—	—	—	0.63 (0.39-1.02)	.06	0.45 (0.27-0.76)	.003
Physical activity^h^ × total stop	—	—	—	—	—	—	1.76 (1.21-2.57)	.003
Stop message^h^ × total stop	—	—	—	—	—	—	3.31 (2.35-4.64)	<.001
Other message^h^ × total stop	—	—	—	—	—	—	1.43 (0.82-2.50)	.21

^a^Hazard ratios for cox proportional hazard models.

^b^Unadjusted model.

^c^Model 1+Total number of stop messages.

^d^Model 2+County RUCC.

^e^Model 3+Interaction term between message type and total number of stop messages.

^f^Not applicable.

^g^RUCC: Rural-Urban Continuum Code.

^h^Interaction term.

**Table 4:** Pairwise comparisons by message type for 2 levels of total stop messages received.

Pairwise comparison	3 stop messages, hazard ratios (95% CI)	6 stop messages, hazard ratios (95% CI)
Other versus nutrition	0.62 (0.44-0.89)	1.84 (0.89-2.55)
Physical activity versus nutrition	0.91 (0.73-1.23)	5.1 (1.68-8.64)
Physical activity versus other	1.47 (1.09-1.88)	2.74 (1.79-3.14)
Stop versus nutrition	15.44 (12.92-18.31)	557.6 (506.33-604.51)
Stop versus physical activity	16.89 (9.61-22.04)	110.1 (76.33-132.63)
Stop versus other	24.84 (12.3-37.5)	303.89 (230.45-374.69)

### Pairwise Comparisons by Message Type

The effect of the number of stop messages differs by SMS text message type (see Table 4). To further explore these relationships, 2 different values representing the 10th and 90th percentile for total number of stop messages received were chosen. These values were used to estimate the hazard ratios for different pairwise comparisons of message types. The effect of the number of stop messages received had differing effects for different pairwise comparisons. Most notably, the impact of the number of stop messages was particularly pronounced when comparing the probability of dropout after receiving a stop message with the probability of dropping out after receiving another type of message. For example, the stop message and nutrition message comparison shows that receiving a stop message had a 15 times greater probability of resulting in a dropout than receiving a nutrition message with 3 stop messages received, but once 6 stop messages had been received, receiving a stop message had a 557 times greater probability of resulting in a dropout than a nutrition message. A similar pattern was observed when comparing stop and physical activity messages as well as stop and other types of messages.

## Discussion

### Principal Findings and Comparison With Previous Work

The objective of this study was to examine the effect of SMS text message type and number of stop messages received on attrition in Text2BHealthy, a text-based health promotion program. We found that overall attrition differed by message type; in particular, sending a stop message substantially increased the risk of participants dropping out of the program compared with other types of messages, including nutrition, physical activity, and others. Although providing information about how to withdraw from a program might be necessary, the way in which this information is provided has important implications for program attrition.

Most participants did not drop out of Text2BHealthy; 85.7% (833/972) were retained through the end of the school year, indicating possible exposure to key messages over a lengthy period. This is consistent with other literature, including 2 meta-analyses that found a mean retention rate of 86% [9] and rates ranging from 46% to 96% [13] across a variety of text-based programs. However, depending on the participant burden in specific program designs, high retention might reflect that passively remaining in a text-based program is easier than taking action to drop out. Future research should move beyond looking at retention to also explore the extent of participants’ active engagement (eg, opening, reading, and acting on SMS text messages received). In addition, program characteristics such as planned program duration, participant burden, frequency of messages, message timing, and difficulty of changing the targeted health behaviors may influence retention. Studies isolating these factors for comparison and meta-analyses that examine program characteristics are needed.

We also found that the median program duration for participants who dropped out of the program was high (102.5 days), meaning that many participants were exposed to a substantial amount of program content despite ultimately unenrolling from the program. This observation is inconsistent with research conducted by Coa and Patrick [14], showing that attrition tends to occur within the first 2 weeks of a program. Although it is unclear why results from these studies differ, program planners should assess the likelihood of attrition at various points in their own programs, take into account when program fatigue is likely to occur when determining program length, and consider overenrolling participants to limit the impact of attrition on program evaluation. SMS text message program formats are both effective in the short term [18,19] and beneficial in extending contact with participants beyond an initial intervention period [20], but more research is needed to examine the conditions under which participants tolerate longer program durations.

In examining the context of program participants, we found that parents living in rural counties were less likely to drop out than parents living in more metropolitan counties. This finding may be explained by the relative scarcity of health services and programs in rural areas. Participants in rural areas may be less likely to drop out of a text-based health promotion program, as rural areas tend to have fewer health services and programs available [15]. It is likely that many other contextual factors may impact attrition, but more research is needed to identify such factors and mitigate their unique impact in SMS text message programs. If limited program availability in rural areas is both a motivation to use SMS text message programs with isolated populations and an explanation of high retention, related characteristics of limited access to resources, health disparities, and isolation such as socioeconomic status, race or ethnicity, immigration status, basic literacy, and exposure to other programs should be examined in future research.

We found that the effect of the number of stop messages received during the program period differs by SMS text message type. In particular, receiving 6 stop messages results in greater probabilities of attrition for all SMS text message types than receiving 3 stop messages, suggesting a possible dose-response relationship between number of stop messages received and the likelihood of dropping out of the program. In addition, the interaction between stop messages and the total number of stop messages received yields the largest effect on attrition compared with the interaction between other message types and the number of stop messages received. Although we were unable to find any other research examining the effect of stop message receipt on attrition, these findings might echo dose-response observations in another study showing that number of messages received was associated with positive behavior changes related to weight management [20]. These findings are also consistent with previous research emphasizing the importance of message characteristics, including content and the number of SMS text messages received, in achieving high retention rates in SMS text message–based health promotion programs [21-23].

Program planners needing to send instructions on how to drop out of a program should consider limiting the number of times this information is provided, as it is possible that a greater frequency of such information has negative implications for program retention. In addition, a better understanding of the ways in which participants’ characteristics impact probability of dropping out after receiving a stop message could also improve retention. In particular, certain participants might be more responsive to stop messages, such as those who join many text-based programs and those with limited facility with SMS text messaging who might drop out accidentally or misunderstand the intention of the stop message. Furthermore, future research may identify the best ways of informing participants about how to remove themselves from the program and illuminate which groups of participants may be expected to already know how to remove themselves from any SMS text message program.

### Limitations

This study has a number of important limitations. First, we were unable to control for individual demographic characteristics that might explain differences in attrition. Second, although the data analysis accounts for different frequencies with which particular messages were sent, the relatively small number of messages combining nutrition content with a stop message and physical activity with a stop message signifies a small number of possible dropout events to observe, resulting in limited or inadequate statistical power. We, therefore, combined these 2 SMS text message types with stand-alone stop messages into 1 group for analysis, which may obscure differences that might have been detected with more observations. It is also possible that certain messages within a message category impacted attrition differentially, but because of somewhat varied content across schools, we were unable to examine attrition probabilities for each unique message. Third, although we are able to link attrition events to the most recently sent message, this does not necessarily indicate that a participant chose to drop out of the program because of the content of this particular message. In addition, program data indicate only that messages were sent to a functioning cellular number, not whether participants read the messages they receive or whether the messages were impactful. Therefore, participant retention is not itself an indication of either participant engagement or program efficacy.

### Conclusions

This study of attrition in the Text2BHealthy program demonstrates the potential of SMS text message programs to retain participants over a long program duration. In examining the patterns of attrition, we have provided evidence that the probability of attrition increases when participants receive SMS text messages with instructions about withdrawing from the program. Program planners should carefully consider how and how often to provide such information to minimize its effect on retention and determine other possible message content and characteristics that may undermine retention. Despite substantial progress in understanding best practices in SMS text message program design and implementation, more research is needed to determine participant, programmatic, and contextual predictors of program duration and attrition to mitigate their impact in SMS text message programs. Furthermore, the relationship between program duration and attrition and targeted behavioral outcomes also necessitates examination.

## Abbreviations

FARM: free and reduced meals
RUCC: Rural-Urban Continuum Codes
SMS: short message service
SNAP-Ed: Supplemental Nutrition Assistance Program-Education

## References

[1] Fjeldsoe BS, Marshall AL, Miller YD (2009). Behavior change interventions delivered by mobile telephone short-message service. Am J Prev Med.

[2] Pew Research Center (2017). Pew Research Center.

[3] Pew Research Center.

[4] Siopis G, Chey T, Allman-Farinelli M (2015). A systematic review and meta-analysis of interventions for weight management using text messaging. J Hum Nutr Diet.

[5] Scott-Sheldon LA, Lantini R, Jennings EG, Thind H, Rosen RK, Salmoirago-Blotcher E, Bock BC (2016). Text messaging-based interventions for smoking cessation: a systematic review and meta-analysis. JMIR Mhealth Uhealth.

[6] Nelson LA, Mayberry LS, Wallston K, Kripalani S, Bergner EM, Osborn CY (2016). Development and Usability of REACH: A tailored theory-based text messaging intervention for disadvantaged adults with type 2 diabetes. JMIR Hum Factors.

[7] L'Engle KL, Mangone ER, Parcesepe AM, Agarwal S, Ippoliti NB (2016). Mobile phone interventions for adolescent sexual and reproductive health: a systematic review. Pediatrics.

[8] Cole-Lewis H, Kershaw T (2010). Text messaging as a tool for behavior change in disease prevention and management. Epidemiol Rev.

[9] Head KJ, Noar SM, Iannarino NT, Grant HN (2013). Efficacy of text messaging-based interventions for health promotion: a meta-analysis. Soc Sci Med.

[10] Speirs KE, Grutzmacher SK, Munger AL, Messina LA (2016). Recruitment and retention in an SMS-based health education program: lessons learned from Text2BHealthy. Health Informatics J.

[11] Herbert L, Owen V, Pascarella L, Streisand R (2013). Text message interventions for children and adolescents with type 1 diabetes: a systematic review. Diabetes Technol Ther.

[12] Gazmararian JA, Elon L, Yang B, Graham M, Parker R (2014). Text4baby program: an opportunity to reach underserved pregnant and postpartum women?. Matern Child Health J.

[13] Siopis G, Chey T, Allman-Farinelli M (2015). A systematic review and meta-analysis of interventions for weight management using text messaging. J Hum Nutr Diet.

[14] Coa K, Patrick H (2016). Baseline motivation type as a predictor of dropout in a healthy eating text messaging program. JMIR Mhealth Uhealth.

[15] Douthit N, Kiv S, Dwolatzky T, Biswas S (2015). Exposing some important barriers to health care access in the rural USA. Public Health.

[16] Grutzmacher SK, Braunscheidel Duru E, Speirs KE, Worthington L, Munger AL, Lachenmayr LA (2017). Using text messages to engage low-income parents in school-based nutrition education. J Hunger Environ Nutr.

[17] United States Department of Agriculture (2016). Economic Research Service.

[18] Bacigalupo R, Cudd P, Littlewood C, Bissell P, Hawley MS, Buckley Woods H (2012). Interventions employing mobile technology for overweight and obesity: an early systematic review of randomized controlled trials. Obes Rev.

[19] Shaw R, Bosworth H (2012). Short message service (SMS) text messaging as an intervention medium for weight loss: a literature review. Health Informatics J.

[20] Spark LC, Fjeldsoe BS, Eakin EG, Reeves MM (2015). Efficacy of a text message-delivered extended contact intervention on maintenance of weight loss, physical activity, and dietary behavior change. JMIR Mhealth Uhealth.

[21] Abroms LC, Whittaker R, Free C, Mendel VA, Schindler-Ruwisch JM (2015). Developing and pretesting a text messaging program for health behavior change: recommended steps. JMIR Mhealth Uhealth.

[22] Hingle M, Nichter M, Medeiros M, Grace S (2013). Texting for health: the use of participatory methods to develop healthy lifestyle messages for teens. J Nutr Educ Behav.

[23] Woolford SJ, Barr KL, Derry HA, Jepson CM, Clark SJ, Strecher VJ, Resnicow K (2011). OMG do not say LOL: obese adolescents' perspectives on the content of text messages to enhance weight loss efforts. Obesity (Silver Spring).

